# An online survey of perspectives towards the impact of the covid-19 pandemic amongst caregivers of adolescents with ASD

**DOI:** 10.1186/s12912-024-02492-w

**Published:** 2024-11-14

**Authors:** Nadlada Tawankanjanachot, Craig Melville, Maria Truesdale, Lisa Kidd

**Affiliations:** 1 School of Medicine, Dentistry & Nursing, College of Medical, Veterinary & Life Sciences, University of Glasgow, Glasgow, G12 9LL UK; 2 Ramathibodi School of Nursing, Faculty of Medicine Ramathibodi Hospital, Mahidol University, Bangkok, Thailand; 3 School of Health and Wellbeing, College of Medical, Veterinary & Life Sciences, University of Glasgow, Glasgow, G12 0XH UK; 4 School of Health & Life Sciences/Research Centre for Health, Glasgow Caledonian University, Glasgow, G4 OBA UK

**Keywords:** COVID-19, Adolescents, Caregivers, Autism spectrum disorder, Thailand

## Abstract

**Background:**

The coronavirus disease (COVID-19) pandemic has had a negative impact on the health and mental health of adolescents and adolescents with autism spectrum disorders (ASD) and their caregivers, have been disproportionally affected. The purpose of this study was to investigate the impact of COVID-19 on Thai caregivers and adolescents with ASD.

**Methods:**

This study used an online survey with closed and free text questions to investigate how the pandemic had impacted on social skills development and psychological variables, and perceived needs for support. The survey link was shared to parents via the Yuwaprasart Withayopathum Child and Adolescent Hospital and the social media platforms of known ASD stakeholder networks in Thailand. Binary logistic regression was used to investigate the relationships between sociodemographic characteristics, service use, and social skills problems experienced by adolescents during the pandemic. Content analysis was applied to analyse free-text data.

**Results:**

A total of 376 caregivers of adolescents with ASD aged 10–19 years completed the survey, of which 364 were included in the analysis. In total, 38.7% of caregivers reported that during the pandemic the social skills of their adolescent family member had worsened. Most families reported that they were able to continue to access support from healthcare and educational services, albeit in different ways than pre-pandemic, during the acute stages of the pandemic which benefited the maintenance of ASD symptoms and social skills. Factors identified as reducing the odds of a worsening in social skills during the pandemic included; regular access to treatment for adolescents (odds ratio [OR] = 0.55, confidence interval 95% (CI) 0.32–0.98, *p* = 0.044), caregivers feeling that they had sufficient support from hospitals (OR = 0.46, 95% CI 0.26–0.81, *p* = 0.007) and older age of adolescents (OR = 0.53, CI 0.29–0.99, *p* = 0.047). The qualitative free text comments showed that the caregivers felt that greater information on managing adolescent behaviours, opportunities for adolescents to practice social skills, and the provision of greater emotional support and material assistance from healthcare professionals during the pandemic would have helped them to care for the adolescents with ASD.

**Conclusion:**

Regular access to services, support from hospitals during the pandemic and older age in adolescence may have helped prevent the worsening of the social skills problems of adolescents with ASD.

## Background

Coronavirus disease (COVID-19) is an infectious disease caused by the SARS-CoV-2 virus. In 2020, the World Health Organization (WHO) declared a global pandemic and a public health emergency of international concern [1]. The majority of those infected with COVID-19 suffer from a mild to moderate respiratory illness and recover without special treatment. However, some individuals become severely ill and even die [2, 3]. Most countries across the world implemented national lockdowns to control the spread of disease by restricting people’s movement, work, education, and gathering [4, 5].

Thailand’s government announced the country’s first national lockdown from 26 March to 30 June 2020, followed by a second lockdown from 12 July 2021 until 31 August 2021 in the greater Bangkok area (Bangkok and five associated provinces). The number of Thai children and adults infected with COVID-19 who suffer from respiratory infections is similar to that of other countries globally [6, 7]. For Thai people, both lockdowns, required people to wear masks, remain inside their homes from 10 p.m. to 4 a.m., and there were school closures and purposeful social isolation [8, 9]. The Thai healthcare service also implemented a comprehensive COVID-19 benefit package (e.g., laboratory tests, vaccination, quarantine measures) without user copayment [9] and effectively managed cases with outpatient self-isolation, home-based isolation, and community-based isolation [10].

Evidence to date shows that worldwide the COVID-19 pandemic had a detrimental impact on the mental health of adolescents [11, 12]. Systematic reviews of the impact of COVID-19 on adolescents reported that those with disabilities were at a higher risk of mental health problems [13, 14], increased mental health symptoms [(e.g., depression, anxiety, stress); [14, 15]] and educational disparities due to a lack of support and resources for remote learning compared to adolescents without disabilities [13]. Similarly, results from a survey conducted by UNICEF [16] in Thailand indicated significant mental health challenges among Thai teenagers. The online survey, which included 6,771 Thai teenagers aged 15–19 years, revealed that 42% of respondents suffered stress as a result of being isolated in their houses, and 47% voiced concern about their ability to continue their studies. Additionally, this suggests that the COVID-19 pandemic may have a differential impact on adolescents and may also result in broader negative effects on the health and wellbeing of adolescents with special needs [17].

For adolescents with ASD, the restrictions that were put in place within society during COVID-19 also had an impact on the severity of adolescents’ symptoms and behaviours [18–22]. Individuals with ASD have deficits in social communication as well as difficulties with various types of behaviour [(e.g. inflexible to routines, difficulty coping with change, difficulty independently organising and planning); [23–25]]. Prior to the pandemic, healthcare for adolescents with ASD in Thailand involved psychosocial intervention, and pharmacological treatment, typically provided through face-to-face sessions in tertiary care settings [26]. Formal group-based therapy (e.g., social skills intervention) was available only in psychiatric hospitals [26, 27]. During the Covid-19 pandemic, however, people were required to socially isolate, causing disruptions to the tightly held day to day routines that helps adolescents with ASD to manage their behaviour and lives. Data shows that such alterations can lead to an increase in behavioural problems [18, 22, 28], a decline in social communication skills [25, 29, 30], and an increase in stress [31, 32] and anxiety in adolescents with ASD [22, 25, 33, 34]. These effect were corroborated by the findings from a UK study which asked caregivers about the effects of the lockdown on the social skills of individuals with ASD [29]. The results suggest that the social communication skills of individuals with ASD deteriorated during lockdown and significantly improved upon their return to school following lockdown periods. Additionally, research has shown that caregivers of adolescents with ASD experienced adverse effects on their mental health [35, 36] and an increased financial burden [37] over the course of the pandemic lockdowns.

In a previous qualitative study, designed to understand the needs of adolescents with ASD and their families towards social skills intervention training programmes, we reported that the closure of schools and some healthcare services in Thailand during the COVID-19 pandemic had an impact on adolescents with ASD and their families [38]. Participants felt that the replacement of face-to-face services with online or telehealth support did not adequately address and meet the needs for support of adolescents with ASD and their parents’ or caregivers’ (hereafter referred to as caregivers). It’s plausible given the growing evidence on the wider impact of the pandemic to suggest that this move to online support, coupled with the closure of schools and limitations on social activities, may have an impact on the social skills of adolescents with ASD [29] and their social and emotional wellbeing [39–41]. Limited evidence currently exists, however, to meaningfully understand the experiences of adolescents with ASD and their caregivers in relation to access to social skills training, healthcare support, and social skills development more specifically. What’s more, studies that have been published to date have largely been conducted in Europe (e.g., the United Kingdom, Italy) and so further exploration of the impact of the pandemic is needed for another context, such as in Thailand. To address this gap an online survey was devised in this study to identify caregivers’ experiences of access to and provision of support and training during the pandemic and their perspectives of the impact of the pandemic on their adolescents’ social skills development during that time. A further aim was to investigate which specific sociodemographic and service factors were associated with the impact of the pandemic on the social skills of Thai adolescents with ASD. This focus provides a valuable lens for directing future research and practice given the changed context of society and healthcare delivery due to the pandemic.

### Research questions

1. What are parents’ and caregivers’ perceptions of the perceived impact of Covid-19 pandemic and associated measures of socio demographics and impact of COVID-19 in Thai adolescents with ASD?
2. What are parents’ and caregivers’ perceptions of service disruptions and provision of and access to social skills training service and healthcare service for Thai adolescents with ASD during the pandemic?

## Method

### Research design

A descriptive online survey was used to collect information from caregivers of Thai adolescents with ASD, aged 10–19. This method was suitable for collecting data during the COVID-19 pandemic when face-to-face interactions were restricted and allowed an opportunity to identify the impact of the pandemic at breadth across a large sample of caregivers in Thailand. The survey was created using online software (Bristol Online Survey; BOS), which allowed for the creation, distribution, and analysis of the survey, and was shared via a link and QR code on the stakeholder network’s social media platforms (e.g., caregivers, healthcare professionals). The survey was available online from January 27 to March 27, 2022. This period marked the fifth wave of COVID-19 in Thailand, caused by the Omicron variant. The pandemic was ongoing during the course of the study, and the Thai government gradually eased restrictions while continuing to monitor cases and respond to outbreaks [8, 42]. The survey was approved by the University of Glasgow (200210052; 5 January 2022) and the local ethics committee of Yuwaprasart Withayopathum child and adolescent hospital (01/2022; 7 December2022).

### Participants

The participants were parents or caregivers of adolescents aged between 10 and 19 years in Thailand who were able to read and understand the Thai language, as the survey was written in Thai. Those participants who did not meet this criteria were excluded from the survey. Participants were recruited via two ways:

1. online advertisements were placed on various social media platforms within caregivers’ networks (e.g., the Autistic Thai Foundation, the Rainbow Room Special Needs Awareness Centre, and the Special Education Centre). These platforms included Facebook, Twitter, websites, and the Line application. The survey link was sent to the webpage administrators, who then distributed it through official communication channels (e.g., Line, email) to parents and caregivers on their contact lists.
2. A poster advertisement was distributed through professional networks (e.g., Yuwaprasart Withayopathum Child and Adolescent Hospital) with the QR code for completing the online survey. The poster and QR code were displayed and shared at the outpatient and inpatient clinics in the hospital. This was deemed a suitable recruitment strategy since this hospital has the highest number of cases of ASD among mental health hospitals in Thailand [43].

The required sample size for this survey was 367, calculated using the Ary, Jacobs [44] study. This calculation was based on a 95% confidence level and a 5% margin of error, considering both the 1,000 members of a webpage parents’ network and the 3,445 adolescents with ASD who received services at the hospital in 2020 [43]. All participants provided electronic informed consent that included information on the study’s objective, procedures, potential advantages of participation and voluntary involvement, and a link to mental health support if they required it. The information sheet was provided on the front page of the survey. Participants clicked “Next” to proceed with the survey or closed the window if they chose not to participate. The survey was confidential, anonymous and did not collect personal data in the forms of names or contact details. All research data was downloaded from the survey platform and saved on an encrypted electronic device and stored on a secure, password-protected computer with access restricted to the research team only.

### Materials

The survey questionnaires were developed based on the available literature at that time [28, 29] and informed from the findings of the earlier qualitative study, conducted by the first author [38]. The survey consisted of 21 questions that asked about:1) participant demographics (e.g., age, gender, region of living, age of adolescent they are caring for, age of diagnosis of adolescent; 9 questions), developed based on literature identifying variables related to social skills in adolescents with ASD [28, 29, 38, 45]; 2) the impact of the pandemic on access to support services [(yes/no question; 8 questions); (adapted from Colizzi, Sironi [28] and 3) perceptions of services and accessibility of social skills intervention during the pandemic (free text questions; 4 questions). The survey took approximately 15 min to complete. Before distributing the survey to the participants, the questionnaires were reviewed by the experts in the wider research team (e.g., psychiatrist, nurse), translated into the Thai language and piloted with a small group of caregivers of adolescents with ASD (*n* = 9) to ensure clarity and suitability of the questions [46, 47]. Only minor modifications (e.g., providing more information on the type of schooling adolescents received e.g., special education) were made, based on the result of the pilot study.

### Analyses

The final raw data from the Bristol Online Survey was downloaded into a Microsoft Excel file and analysed using SPSS software (version 25). Quantitative analyses comprised descriptive statistucs and logisitc regression analyses to address the first research question. Descriptive statistics were used to illustrate the sociodemographic information and impact of COVID-19 among adolescents with and without new or worsening social skills problems during the pandemic. Binary logistic regression was used to investigate the association between social skills problems (the dependent variable) and sociodemographic and service factors. The variables included in the logistic regression analyses included (i) socio-demographic variables: ages of caregivers, region of living, age of adolescents, attendance at school, and ability of communication. (ii) service variables (yes/no; attendance at private therapy, adolescents’ regular access to treatment, sufficient support from hospitals, sufficient access to online treatment, regularly attending school, sufficient support from schools, and caregivers sufficient support from the hospital and getting online treatment.

The bivariate analyses between were conducted using the chi-square test to examine the relationships between social skills problems and sociodemographic and service variables. Only variables that tested significant with a p-valve of < 0.25 were taken forward to the multivariate analysis [48, 49]. Using p-value < 0.25 ensure potentially important predictors, including confounders or interactions, are not prematurely excluded before multivariate analysis [48, 49]. Multivariate analysis was performed to examine which variables were independently associated with the social skills problems. Backward stepwise procedure was used to eliminate variables that were not significant (Wald statistic p 0.05). The overall fit of the final main effect model was assessed by the Hosmer-Lemeshow goodness of fit statistic- a large p-valve (*p* > 0.10) indicates a good fit of the model relevant to the data. The assumption of multicollinearity was also assessed (tolerance > 0.10 and Variance Inflation Factor (VIF) < 10) [50]. The final model was assessed to ensure it met the assumptions of logistic regression.

The open-response questions were treated as qualitative data and were used to identify caregivers’ experiences of accessing and receiving support and training during the pandemic, addressing the second research question. The open text responses were analysed using content analysis [30, 51, 52], which followed three steps. The first step was familiarising the data. This was conducted by NT through reading and re-reading the transcripts to identify initial ideas, potential codes and categories that could be constructed from the data. The second step involved the formulation of codes. This was conducted by NT, highlighting where pieces of text conveyed a specific idea or made a contribution towards the overall analysis. The initial coding framework was then applied across all of the transcripts. The third step involved ‘pooling’ of the codes to form categories that helped to provide an overall synthesis of the data. This was conducted by NT and discussed and refined with co-authors (LK, MT). There were no disagreements during the discussion of the codes and categories.The final content analysis consisted of two categories (e.g., the impact of pandemic on adolescents with ASD and caregivers and access to and provision of services for social skills training).

## Results

A total of 376 caregivers participated in the survey. There was missing data for 12 participants in response to the question about the age of the adolescent. These participants were therefore excluded from the final analysis. Following their exclusion, the dataset contained no missing data, and all variables were complete for the analyses. The final analysis included 364 participants.

### Description of sample characteristics

Demographic information on caregivers and their adolescents is presented in Table 1. The caregivers included 288 (79.1%) females and 76 (20.9%) males. The majority of the caregivers were older than 40 years old (82.7%). Participants with ASD lived across six regions: 79.1% lived in the central region, and 20.9% lived in the other regions (North, East, Northeast, South, and West). The majority of adolescents were in early adolescence (10–14 years old; 48.6%). Most caregivers had known of their child’s diagnosis for 2 or more years (34.6%). 83% of the adolescents attended school during the pandemic. Nearly 30% of the adolescents’ participants attended primary school; 29.1% attended secondary school; 22.8% attended special education/community learning centres; and 1.4% attended a vocational diploma programme. 90% of the adolescents with ASD were reported as being able to communicate verbally. It was reported that almost 60% of adolescents did not receive other private therapy.

**Table 1:** Socio-demographic and clinical characteristics of caregivers and adolescents with ASD

Variables	Categories	Frequency	%
Caregivers			
**Gender**	*Female*	288	79.1
	*Male*	76	20.9
**Age (in years)**	*less than 40*	63	17.3
	*More than 40*	301	82.7
**Adolescents with ASD**			
**Region of Thailand**	Central-Region	288	79.1
	Non-Central region	76	20.9
	*- North*	*26*	*7.1*
	*- East*	*19*	*5.2*
	*- Northeast*	*16*	*4.4*
	*- West*	*3*	*0.8*
	*- South*	*12*	*3.3*
**Age of adolescents (in years)**	*10–14*	177	48.6
	*15–17*	116	31.9
	*18–19*	71	19.5
**Duration since diagnosis**	*Less than 1 year*	17	4.7
	*1 year*	26	7.1
	*2 years*	126	34.6
	*3 years*	109	29.9
	*4 years*	47	12.9
	*+ 5 years*	34	9.3
	*Not mention*	5	1.4
**Attending school during the pandemic**	*Yes*	302	83.0
	*No*	62	17.0
**Grade of study**	*Primary (10–12)*	108	29.7
	*Secondary (13–17)*	106	29.1
	*University (18–19)*	5	1.4
	*Special education/ Community Learning Centre*	83	22.8
**Ability of verbal communication**	*Yes*	332	91.2
	*No*	32	8.8
**Attending private therapy**	*Yes*	154	42.3
	*No*	210	57.7

### Perceptions of the impact of Covid-19 and associated measures of impact

During the lockdowns, caregivers reported that 141 (38.7%) adolescents with ASD reported new or worsening social skills problems, highlighting the perceived impact of the pandemic on the social development of adolescents with ASD. Table 2 reports the bivariate analysis examining which variables were associated with new or worsening social skills problems. A p-value threshold of < 0.25 was used in this initial stage to screen variables for their potential relevance to social skills problems (the dependent variable).

Variables associated with social skills problems (*p* < 0.25) included living outside the middle region of Thailand, age of adolescents, non-attendance at school, adolescents not regularly accessing treatment, adolescents receiving insufficient support from hospitals and accessing online treatment, caregivers receiving insufficient support from hospitals and caregivers receiving online treatment, adolescents not regularly attending school and receiving insufficient support from school. These associated variables were included in the multivariate analysis to better understand their combined influence on the social skills of adolescents with ASD during the pandemic. There was no association between parents’ gender, ability of verbal communication, and receiving private therapy with social skills problems.

**Table 2:** Sociodemographic, the impact of COVID-19 characteristics of participants with and without social skills problems

Variables	Participants with social skills problems (*n* = 141)	Participants without social skills problems (*n* = 223)	Odd Ratio (95%CI)	*P* valve
	Frequency (%)	Frequency (%)		
***Sociodemographic characteristics***				
**Caregivers**				
**Gender**				
Male	32(22.7)	44(19.7)	REF	0.498
Female	109(77.3)	179(80.3)	0.837(0.501,1.400)	
**Age (in years)**				
Less than 40	24(17)	39(17.5)	1.033 (0.591, 1.807)	0.909
More than 40	117(83)	184(82.5)	REF	
**Adolescents with ASD**				
**Region of Living**				
Middle	106(75.2)	182(81.6)	1.466 (0.880, 2.443)	0.142
Non-middle	35(24.8)	41(18.4)	REF	
**Age of adolescents (in years)**				0.004
10–14	76 (53.9)	101(45.3)	REF	
15–17	40(28.4)	76(34.1)	0.699(0.431, 1.136)	0.149
18–19	25(17.7)	46(20.6)	0.722(0.408, 1.278)	0.264
**Attending school during the pandemic**				
No	28(19.9)	34(15.2)	REF	
Yes	113(80.1)	189(84.8)	0.726(0.418, 1.261)	0.255
**Ability of verbal communication**				
No	18(12.8)	14(6.3)	REF	
Yes	123(87.2)	209(93.7)	0.458(0.220, 0.953)	0.37
**Attending private therapy**				
No	90(63.8)	120(53.8)	REF	
Yes	51(36.2)	103(46.2)	0.660 (0.428–1.018)	0.60
***Reported impact of the COVID-19***				
**Adolescents regular access to treatment**				
No	58(41.1)	45(20.2)	REF	
Yes	83(58.9)	178(79.8)	0.362(0.226, 0.578)	< 0.001
**Adolescents receiving sufficient support from hospital**				
No	47(33.3)	35(15.7)	REF	
Yes	94(66.7)	188(84.3)	0.372(0.225, 0.616	< 0.001
**Adolescents sufficient access to online treatment**				
No	86(61)	106(47.5)	REF	
Yes	55(39)	117(52.5)	0.579 (0.377, 0.885)	0.013
**Caregivers receiving sufficient support from hospital**				
No	68(48.2)	52(23.3)	REF	
Yes	73(51.8)	171(76.7)	0.326(0.207, 0.514)	< 0.001
**Caregivers received online treatment**				
No	70(49.6)	77(34.5)	REF	
Yes	71(50.4)	146(65.5)	0.535 (0.348, 0.823)	0.004
**Adolescents regularly attending school**				
No	65(46.1)	86(38.6)	REF	
Yes	76(53.9)	137(61.4)	0.734(0.479, 1.125)	0.156
**Adolescents receiving sufficient support from school**				
No	69(48.9)	68(30.5)	REF	
Yes	72(51.1)	155(69.5)	0.458(0.296, 0.708)	< 0.001

The final multivariate model is shown in Table 3. Only three factors were significantly associated with worsening of social skills problems during the pandemic and hence, were retained in the final model.

1. older age of adolescents [(aged 18–19 years); (odd ratio (OR) = 0.53, 95% confidence interval [CI] 0.29 to 0.96)]
2. regularly accessing treatment (OR = 0.56, 95% CI 0.32 to 0.99).
3. caregivers’ receiving sufficient support from the hospital (OR = 0.46, 95% CI 0.27 to 0.81).

The logistic regression model was statistically significant, χ2 [8] = 38.956, *p* = 0.000 and explained 13% (Nagelkerke R^2^) of the variance in social skills problems and correctly classified 69% of cases. Hosmer and Lemeshow’s test for the final model was 0.213, which indicated a good fit model (*p* > 0.010). All three predictors (older age of adolescents, regular access to treatment, and caregivers receiving sufficient support) are associated with a reduced likelihood of the outcome. The odds ratios and their corresponding confidence intervals suggest that these associations are statistically significant, indicating that interventions targeting these factors may help decrease the risk of the outcome.

**Table 3:** Final multivariate logistic regression model for the association between sociodemographic and the impact of the COVID-19

Variables	B	S.E.	OR	95%CI	*P*-value
**Age of adolescents**					
− 10–14 years					
− 15–17 years	-0.40	0.26	0.67	0.40 to 1.11	0.126
− 18–19 years	-0.63	0.31	0.53	0.29 to 0.99	0.047^*^
**Adolescents regular access treatment**	-0.58	0.29	0.56	0.32 to 0.99	0.044^*^
**Caregivers sufficient support from hospital**	-0.77	0.28	0.46	0.27 to 0.81	0.007^**^

(SE; standard error, OR; odd ratio, CI; confidence interval; * P-valve < 0.5, **P-valve < 0.005)

### Parents’ and caregivers’ perceptions of service disruptions provision of and access to services for social skills training and healthcare

The responses to the open questions were analysed and reported on as below. Two categories were identified from the content analysis: [1) Access to and provision of services for social skills training and health, 2) Access to and provision of services for social skills training and health]. These are reported on in the following sections with exemplar quotes were provided in each category.

### The impact of pandemic on adolescents with ASD and the caregivers

According to the caregivers, the pandemic had negative impacts on them and their adolescents including.

#### Social skills, behaviour, and development of adolescents with ASD

Due to the restrictions, hospitals had to postpone services and treatments. Caregivers perceived that this had influenced the development and behaviour of the adolescents. For example, they observed more aggressive behaviour. In addition, due to a decline in outdoor activities, interaction with other people, and schools closing, many adolescents’ social skills changed, and many started using social media heavily.

*“Due to COVID-19*,* a lot of treatment appointments were cancelled. My child has lost his training session. I want some activities to reduce his emotional behaviour."*

"*His social development regressed. I want some solution."*

Several of the participants displayed increased levels of self-injurious behaviour during the pandemic, which caregivers attributed to a lack of medication and the adolescents’ training.

*"At home*,* my child hurts himself*,* but we cannot go to the hospital because of lockdown. He’s very upset. I want to visit a doctor to get some medicine treatment*."

Additionally, they were distressed by the prospect of being quarantined at home.

#### Loss of face to face opportunities for education

Adolescents with ASD received online schooling rather than onsite, face to face schooling throughout the pandemic. Some of adolescents were reported as being consumed by schooling, having been used to a face-to-face model, and found it more difficult to engage in independent self-study and organisation of their work. As a result, they may have increased their repetitive behaviour due to difficulties in changing their routine, according to the free text data.

*“When the COVID-19 restrictions are lifted*,* I want the school to provide some on-site classes because my son keeps asking repeatedly [repetitive behaviour] to go to school.”*

#### Anxious about infection

The caregivers revealed that their lack of knowledge regarding disease prevention for COVID-19 made it difficult to care for adolescents with ASD. Some adolescents were more anxious due to their fear of contracting diseases. In addition, it was challenging to find a hospital that would accept COVID-19 patients with ASD because several institutions lacked the required staff capabilities.

*“If our children get an infection*,* I [parent] ask for some urgent help. It is quite difficult to find a specific hospital for such autistic children”.*

#### Financial impacts

Caregivers reported that they had been unable to work normally since the pandemic began. Some caregivers were unemployed and without income which had a financial impact on them.

*“I [parent] was stressed and couldn’t work well*,* which led to me being fired.”*

*“They[parents] are now out of work and have no income.”*

### Access to and provision of services for social skills training and health

#### Healthcare and education setting accessed during the pandemic

The caregivers mentioned the healthcare and education services to which adolescents and themselves had access, including public and private hospitals, community and school networks, and special services (Table 4).

**Table 4:** Caregivers-reported information of service that the adolescents and themselves can access

Services accessed during the pandemic	For Adolescents (*n*)	For Caregivers (*n*)
**Government healthcare service** (Child psychiatric hospitals/ Region-General hospital/ Community hospital/ University hospital)	178	111
**Community Network service** (Parent’s network/ Centre of Autism Empowerment/home(online)/ temple)	50	17
**Private healthcare service** (Private hospital/clinic)	27	13
**Education service** (School/Special education centre)	19	7
**Special service for COVID-19** (Field hospital)	3	-
**No access to any services**	52	39
**No response**	80	43

During the lockdown, the government healthcare service was the most frequently used service (e.g., public hospitals). Community network services (e.g., parents’ network) were the second-most accessed services. The third service utilised was the school’s network, which includes schools and education centres across the province. The least utilised service was the COVID-19 special service (e.g., field hospital). Nearly fifty participants reported that they could not access the healthcare services, and between forty and eighty individuals did not respond. It can be observed that the proportion of individuals accessing healthcare and educational services is higher than the population unable to access these services.

#### Services access and provision for social skills training and health

Most caregivers perceived a need for continuous care for adolescents with ASD during a pandemic, including.

**Continuous care**.

##### 1) Telemedicine

Many caregivers supported adolescents to attend follow up appointments with the psychiatrist via phone or video call in order for the psychiatrist to assess their development and behaviour, provide advice, and monitor their medication. This support enabled continuous care during the pandemic, with online follow-ups possibly becoming more common than in-person visits. The caregivers also required a social media channel (e.g., The LINE application), which makes it easy to communicate with the health care professional.

*"HP should provide some communication channels through which we can ask for suggestions or help. If we need some medicine or a suggestion of a symptom*,* there should be a service centre that uses LINE or VDO. So that we can send the child’s updated photo and a VDO clip of our child’s behaviour. We can access this communication channel immediately."*

##### 2) Medication delivery

Caregivers require home delivery medicine to keep adolescents’ symptoms stable.

"*Provide medicine via postal service so that we can get the treatment continuously."*

##### 3) Learning materials

Caregivers required learning materials for both them and adolescents with ASD. They indicated that the most advantageous options are the online materials and the home-delivered boxes of materials. The online material could be easily accessed, allowing for independent practise and providing visual examples. For instance, the videos and activities enhance some areas of social skills. Caregivers mentioned the required topics of knowledge: how to deal with adolescents as they transition into adolescence; activities that can help reduce behavioural problems; and how to teach social and daily life skills.

"*I want some VDO clip, not only the paperwork for parents so that we could train our children at home."*

*“We want continuous therapy. If we cannot go to the service centre*,* we would like to get some suggestions or learning material boxes at home.*

The caregivers perceived that adolescents could attend additional lessons and be provided with learning materials during the quarantine, and that there would be a return to onsite, face to face schooling once the pandemic was under control.

"*I want some additional study classes because he lost a lot of time studying. A child should get some academic paperwork from school."*

"*During the COVID-19 situation, I want the school to provide some on-site classes because those children [adolescents with ASD] keep thinking [obsessive] that they want to go to school."*

##### 4) Counselling

Since the pandemic, adolescents have exhibited a rise in behavioural issues, according to caregivers. Caregivers and adolescents with ASD were stressed. They needed the counselling hotline. It may be a video call for counselling, during which caregivers would be able to display photographs or video clips of adolescents’ symptoms.

"*I want a VDO call channel that provides me with the suggestion. Thus, I can get feedback from HP."*

##### 5) On-site Medical care

Some adolescents were unable to receive treatment online. For instance, adolescents who were unable to communicate effectively may have had difficulty participating in certain treatments (e.g., group activities). Subsequently, adolescents required treatment and training on-site in an environment safe from COVID-19.

*“I ask for some suitable group training under the COVID-19 regulation.”*

"*There are some limitations to the online treatment service because my child doesn’t understand."*

##### 6) Home visits

The caregivers indicated that if they were unable to travel to the hospital, they required home healthcare visits for assessment, development, and skill training. In addition, the caregiver is exhausted and in need of rest. As a result, they suggest that it could have a foster home that could occasionally care for adolescents with ASD.

"*I want some staff to visit my home and provide 2–3 h of training at home."*

"*I want an autism child’s nursery so that I can take some rest."*

##### Financial support

The caregivers reported relying on the government for education funding as well as assistance with finances, food, and medicine. They also reported needing education and a vocational path (e.g., an online marketplace) for them and adolescents.

*“If there is some help*,* I want the government to support some careers for those autism children’s parents. I am now out of work and have no income.“*.

*“I want some material for agriculture work like seed*,* soil*,* mushroom lumps*,* and an online market for those autism children’s parents.“.*

#### Prevention of COVID-19 and service support

The caregivers recommend the organisation of a pandemic-specific health care system for an individual with ASD. For instance, a specialised ward for admission when adolescents contracted COVID-19. They also needed specific knowledge for caring for an individual with ASD (e.g., prevention techniques, home isolation), protective equipment (e.g., face masks, lateral flow tests), and telemedicine to monitor adolescent’s symptoms.

"*I want a long-term care plan to deal with the COVID-19 situation, such as caring protocols, online practising, how to teach our children to prevent the spread of the virus, and free face mask covering."*

*“We want to get fast track*,* special ward treatment for these autism children. Parents can also stay with him.*

## Discussion

This online survey was undertaken to determine the perspectives of caregivers regarding the influence of the COVID-19 pandemic (during the fifth wave of COVID-19 in Thailand), accessibility of services, the need for support, and the sociodemographic factors associated with worsening of social skill problems in Thai adolescents with ASD. The overall findings were that the pandemic had impacted negatively upon adolescents with ASD and their caregivers. Furthermore, during the pandemic, older age, regular access to treatment from adolescents and support provision for caregivers were all associated with a lower odd of the adolescents developing new or worsening social skills problems. The findings serve to reinforce that caregivers require greater information, emotional support, and material assistance from healthcare professionals, which encourages them to care for their adolescents.

### Perception of impact during the COVID-19 pandemic

In this study, the pandemic impacted caregivers and adolescents with ADS’ social skills and behaviours, mental health, and education. The study findings support the previous studies that have highlighted the pandemic’s effects on social skills in children and adolescents with autism [25, 28, 29, 53, 54]. Caregivers of children with ASD in China reported that 36.2% of their children emotional had experienced a worsening of their social skills [53]. Additionally, our free text data revealed an increase in behaviour problems and anxiety among Thai adolescents with ASD during the COVID-19 pandemic. This finding is consistent with Hamilton, Kelly [25], who interviewed British adolescents with ASD (*n* = 13, aged 13–14 years) during the pandemic, revealing increased stress, loss of social relationships, disrupted learning, and heightened anxiety about the virus [25].

### Sociodemographic variables associated impact of COVID-19

The findings identified that protective sociodemographic factors against the worsening of social skill problems in Thai adolescents with ASD included adolescents having regular access to treatment, caregivers receiving regular support for them in their roles, and adolescents being older in age. According to the findings of our study, the first protective factor for preventing the worsening of the social skills problem in adolescents with ASD was continuation of adolescents’ regular access to treatment. Our free text data showed that adolescents with ASD were able to access adaptive treatment such as telemedicine via phone follow-up with psychiatric professionals during the pandemic. This finding was similar to Logrieco, Casula [54], who discovered that receiving treatment during the first lockdown (e.g., Applied Behaviour Analysis (ABA), speech therapy, and parent training via telehealth) is a protective factor for adolescents with ASD’s social interaction, ASD symptoms, and behaviour. However, survey participants reported that only follow-up telemedicine is not sufficient for caring for adolescents’ social skills and behaviours. The literature acknowledges that healthcare providers should provide varied treatment [55], such as medication [34], learning material [40, 56], counselling [57], and home visits. The Secret Agent Society [(SAS; [58]] and the Program for the Education and Enrichment of Relational Skills [(PEERS; [59]] are examples of modified social skills intervention that have been adapted to be delivered via an online platform. This adaptation of online PEERs in the Polish version showed the efficacy of improving social skills and interactions with peers in Polish adolescents with ASD (aged 11–16; *n* = 29) during COVID-19 restriction [(effect size > 0.14); [60]]. These adaptive treatments could be used to develop care services to improve the social skills of adolescents with ASD and their families in the future by integrating the online platform as a training session and homework into the programme.

The second protective factor in preventing social skills problems in adolescents with ASD was caregivers’ access to adequate hospital assistance. Our survey results highlighted that the provision of continuous, healthcare professional-led care and support (e.g., follow-up, treatment) for caregivers could reduce stress in caregivers [18] and subsequently a protective factor of worsening social skills problems in adolescents with ASD. Specifically, treatment for caregivers could include knowledge enhancement (e.g., social skills training, behaviour modification, and preventing COVID-19), video training, a video call follow-up by a doctor, counselling, and medication delivery, according to free text comment. Similarly, Stadheim, Johns [61] interviewed American’ parents with ASD (aged 3–18 years, *n* = 122). The findings revealed that parents who experienced loss of support (e.g., usual healthcare support, knowledge to help their children cope with the pandemic) had an impact on children and adolescents with ASD’s regression on social communication. Additionally, previous studies showed that many caregivers of adolescents with ASD experienced mental health problems due to the adolescents’ behaviour and social skills problems during the pandemic [37, 62]. Caregivers also face numerous challenges in the transitional delivery of home-based education and training for their adolescents [63, 64]. This result led to strengthening the role of parents in coaching and caring for their adolescents.

The final protective factor for preventing social skills problems during the pandemic was late adolescence. This result may be based on social development. The previous study of Sumter, Bokhorst [65] found that late adolescence has less peer pressure, and fewer worries about entering adulthood than younger ages [66]. However, McMahon, Vismara [67] reported factors affecting the improvement of social skills in adolescents with ASD depending on different characteristics such as age, sex, and cognitive ability. Thus, future studies are needed to further explore the protective capacity of older adolescence and the factors that influence this.

However, our findings were different in the delivery of support from those of Morris, Hope [29] and Colizzi, Sironi [28], who discovered that support from school was significantly associated with social communication change in British (aged 3–12; *n* = 176) and Italian (mean age of 13; *n* = 529) individuals with ASD during the pandemic. These different results showed that, based on our free text comments, some adolescents were unfamiliar with the delivery of online classes and required extra support. Furthermore, the Thai education sector experienced difficulties establishing an online and distance learning system during the pandemic [68]. Furthermore, many Thai students face challenges with a lack of digital devices and illiteracy [68]. Hurwitz, Garman-McClaine [69] suggested that the key to successfully educating individuals with ASD during the pandemic was to collaborate with the parents to deliver the online schooling and set the specific goal of teaching with them. In the future, this strategy might be effective when implementing the online class with adolescents with ASD.

The variables that were not associated with social skills problems in Thai adolescents with ASD during the COVID-19 pandemic included a lack of attendance in private therapy, ability of verbal communication and the parents’ gender. Free-text responses supported the finding regarding private therapy, highlighting the role of government hospitals and community networks in providing continuous care during the pandemic. This may be because a large number of participants in our study were from the central region of Thailand, which offers more mental healthcare services compared to other regions, as well as financial constraints [70, 71]. The lack of association between adolescents’ verbal communication abilities and social skills problems may be attributed to the broader components of social skills and had environments factors during the COVID-19 pandemic. Additionally, the absence of a relationship between parents’ gender and adolescents’ social skills problems could be due to the equal involvement of both parents during the pandemic, with both genders having access to healthcare services.

### Perception of access service and provision of social skills intervention

Caregivers and adolescents perceived that they received the continuous care and needed specific support on pandemic information and special services, caring for their adolescents’ symptoms, emotional and learning material support from health providers was important [53]. Similar to our results, previous studies also mentioned that the needs and of supports for the adolescents and caregivers during the pandemic, including visual information for teaching autistic individuals to prevent and deal with COVID-19 [72], caregiver and family training, social and emotional support [73], and financial support [73]. This support can decrease the mental health problems of caregivers [37, 73] and adolescents’ severity of behaviour and social skills [39, 72].

Telehealth was and remains a potential treatment for adolescents with ASD and their caregivers during the pandemic. This type of treatment is appropriate when the health care system significantly restricts in-person delivery [74], increasing treatment availability [75, 76], and saving cost for patients [76, 77]. There was an increase in adapting online delivery techniques to improve social skills for adolescents with ASD during the COVID-19 pandemic, such as the PEERS [60]] and the Secret Agent Society Group Social Skills Programme [58]. Approximately 60% of participants in this study received online treatment such as telemedicine and individualised learning materials from healthcare providers.

Nevertheless, the free text responses in the survey implies that this treatment may not be sufficient to support all adolescents with ASD and their caregivers in a variety of areas, and that certain materials may not be appropriate for them [32]. These results indicate the efficacy of telehealth for individuals with ASD is variable [74, 78]. Additionally, Thai culture and personal beliefs may influence the success of telehealth implementation. A study by Thanakijsombat, Bhatiasevi [79] found that Thai participants in rural areas were uncomfortable using telehealth systems, preferring in-person consultations. In the future, before implementing the programme, healthcare providers should evaluate limitations such as the ability to use the online platform, cultural factors and provide instructions for using the online sessions. Policymakers could also support this effort by improving infrastructure, providing financial assistance, and offering digital literacy training for staff.

To provide telehealth for adolescents with ASD, healthcare professionals may consider patient ethics, equality, and the necessity and barriers to utilising this treatment [80, 81]. The World Health Organization and International Telecommunication Union [82] also suggest telehealth guidelines for individuals with disabilities. These telehealth services could use simple language, provide user instructions, offer convenient access methods (email, SMS, phone, or online), and accommodate various learning limitations (for example, for children). These requirements may result in increased telehealth efficacy for adolescents with ASD and their caregivers in the future.

Parents played a crucial role in providing ongoing care for adolescents with ASD during the COVID-19 pandemic, helping to prevent social skills deficits and behavioural problems in these adolescents. The findings of our study align with the concept of family-centred care (FCC) [83, 84]. This framework emphasises collaboration with parents in delivering care, sharing information, and providing support in areas such as education, psychosocial, emotional, and financial needs [85]. This approach can improve clinical outcomes [86, 87] and enhance the continuity of care [88]. In the future, nurses could implement FCC to provide continuous care for families of adolescents with ASD. This would involve empowering parents in their role, providing education and emotional support, and imparting knowledge on managing ASD symptoms. Furthermore, integrating telehealth into this model could enhance the quality of support.

### Strengths and limitations

To our knowledge, this is the first study to investigate the impact of the COVID-19 pandemic and associated worsening social skills problems on Thai adolescents with ASD. The strengths of this study included the use of both quantitative data and free text data, providing more comprehensive information about the need for healthcare support and social skills training during the pandemic. This can inform the design of provision social skills training programmes. The online survey allowed reach to a large number of people, cost savings, and facilitated a quick response time. Nonetheless, generalising our findings should be done with caution due to the nature of online surveys, which are designed to protect respondent anonymity, do not utilise software tracking and only capture the responses of people who choose to respond. Furthermore, it would have been useful to be able to probe for depth and understanding in some of the responses from participants and to gain an understanding of the experiences of people who chose not to respond to this survey; an important implication for future research. One limitation of this study is the incomplete free-text responses from participants, with 34.57% either accessing healthcare services (13.3%) or not providing any response (21.2%). This could affect the results. Future studies could revise the questionnaire to identify the reasons for non-response [89].

Although the data from this study is two years old, these insights remain valuable for shaping future social skills training programs to effectively meet current needs. Furthermore, the majority of participants lived in the central region of Thailand and were caregivers of adolescents with ASD. It is important to note that Thai culture varies across different regions (e.g., lifestyle) [90]. Future research should aim for a larger sample size from participants in diverse regions and include surveys of adolescents with ASD who are directly involved in social skills intervention programmes.

### Implications for practice, policy and research

According to the findings, receiving regular support and input from existing services, even if provided in different formats to non-pandemic times, is a protective factor in avoiding the worsening of social skills problems during situations like a worldwide pandemic. During the pandemic, Thailand’s healthcare system transitioned from onsite to telehealth treatment delivery. This type of treatment was viewed favourably by participants in this survey for increasing and maintaining consistency of the accessibility of treatment during this time. Because of the pandemic situation, it’s likely that moves towards telehealth for providing access to services and support for adolescents with ASD and their caregivers have been accelerated and become common place in practice, increasing opportunities for better support for people as a result. Where services have returned to face to face formats, the experiences of this pandemic will be useful for facilitating a rapid shift to, and implementation of, telehealth and remote services if necessary in future pandemics. In a sense, services should now be telehealth-ready if required. Further work, however, would be useful around preferred formats of telehealth, how the content of this needs to be tailored towards specific needs and priorities, and the development of knowledge and skills, and guidelines to be able to navigate the use of technology in general. These aspects would have important implications for policy makers.

Nurses play a key role in collaborating with multidisciplinary teams and families to deliver care for adolescents with ASD and their caregivers. Based on the FCC framework and our study, services for adolescents can be enhanced by strengthening the role of caregivers, providing support in education, emotional well-being, and information, as well as training nurses who are not specialists in adolescent ASD care. These efforts could improve clinical outcomes and accessibility for adolescents with ASD and their caregivers.

## Conclusion

In conclusion, the pandemic of COVID-19 has had a negative impact on adolescents with ASD and their caregivers in Thailand. During the pandemic restrictions in Thailand, caregivers observed a worsening of adolescents’ social skills. Our findings indicate that regular access to services, adequate hospital support for caregivers, and older adolescent age prevented worsening social skills during the pandemic. The adaptability of telehealth’s has enabled healthcare professionals to provide continuous care during the pandemic as much as possible and has important implications for the ways that current services could be delivered from hereon in. Thus, the acceleration in service redesign and organisation and provision of care during the pandemic has important implications for practitioners, health service providers and policymakers. Future research would be useful to understand how solutions such as telehealth, for example, could be tailored more towards the needs and priorities of caregivers and adolescents with ASD, particularly in addressing their medical needs. These findings and requirements could be used to reform the treatment and online social skills training programme for adolescents with ASD, ensuring continuous access during lockdowns and pandemics, leading to improved outcomes and quality of life.

## Data Availability

Due to ethical and legal restrictions concerning the confidentiality of study participants, the generated and analysed datasets are not available to the public.

## Abbreviations

ASD: Autism Spectrum Disorder
ABA: Applied Behaviour Analysis
PEERS: Program for the Education and Enrichment of Relational Skills
